# Predictors of Spontaneous Preterm Birth Among Pregnant Women With a History of Cervical Conization

**DOI:** 10.7759/cureus.91558

**Published:** 2025-09-03

**Authors:** Takuma Yamada, Naruhiko Takahashi, Ryo Yamamoto, Shusaku Hayashi

**Affiliations:** 1 Maternal Fetal Medicine, Osaka Women's and Children's Hospital, Izumi, JPN; 2 Maternal Fetal Medicine, Osaka Women’s and Children’s Hospital, Izumi, JPN

**Keywords:** cervical conization, cervical length measurement, high-risk pregnancy, in vitro fertilization, spontaneous preterm birth

## Abstract

Objective

To explore the predictors of spontaneous preterm birth (PTB) among pregnant women with a history of cervical conization.

Methods

This retrospective cohort study included pregnant women with a history of conization who visited a tertiary perinatal center before 20 weeks of gestation between 2013 and 2022. Exclusion criteria were cerclage before 24 weeks of gestation, premature rupture of membranes or delivery before 24 weeks of gestation, multiple pregnancies, fetal death, and major fetal anomalies. Multivariate logistic regression analysis was performed to examine the associations between spontaneous PTB before 37 weeks of gestation and variables, including previous spontaneous PTB or second-trimester loss, in vitro fertilization (IVF), pre-pregnancy body mass index, current smoking status, and mid-trimester cervical length (CL). The spontaneous PTB rates among groups stratified by independent predictors were calculated.

Results

The final analysis included 119 women. Of these, 16 (13.4%) had spontaneous PTB. Multivariate logistic regression analysis revealed that IVF [adjusted odds ratio (OR) = 4.5; 95% confidence interval (CI): 1.2-15.9; p = 0.021] and CL (adjusted OR per 1 mm increment = 0.88; 95% CI: 0.82-0.94; p < 0.001) were significant predictors of spontaneous PTB. The incidence of spontaneous PTB in women who conceived by IVF and/or had a shortened CL<25 mm was 31.1% (14/45), whereas women who did not undergo IVF or had a shortened CL had a lower incidence of 2.7% (2/74).

Conclusion

Conception by IVF and a shortened CL are significant predictors of spontaneous PTB in women with a history of conization.

## Introduction

A history of cervical conization is associated with preterm birth (PTB). According to a meta-analysis that included 53 observational studies, the incidence of PTB was 11.2% among pregnant women who underwent excisional treatment for cervical intraepithelial neoplasia before pregnancy, with a relative risk of 1.87 [95% confidence interval (CI): 1.64-2.12] compared with non-treated women [1]. Recently, a large-scale registry-based study in Japan reported a higher incidence of PTB (25.3%) in pregnant women with a history of cervical conization [2]. This finding highlights the importance of effective prediction and prevention of PTB in these women.

Screening of a short cervix using transvaginal ultrasonography in the mid-trimester has been shown to be an effective predictive method for PTB in both low and high-risk populations [3]. Several studies have assessed the association between cervical length (CL) and PTB in pregnant women with a history of cervical conization, and most have confirmed this association [4,5]. With a cutoff value of 25 mm, which is commonly accepted irrespective of the risk factors for PTB, the reported sensitivities of a shortened CL to predict PTB in women with a history of conization ranged from 41-75% [4,6]. This means that one-half to one-fourth of the PTBs were unpredictable using CL measurements alone. Risk stratification by CL and additional independent predictors may improve the identification of at-risk women compared with stratification solely by CL [7].

Predictors of PTB in women with a history of cervical conization have not been extensively investigated, except for some surgical variables and mid-trimester CL [1,8]. We hypothesized that CL and other maternal characteristics, such as in vitro fertilization (IVF) conception, could predict spontaneous PTB among women with a history of cervical conization and thereby inform clinical management strategies for women with a history of cervical conization. In this study, we explored the predictors of spontaneous PTB in women with a history of conization.

## Materials and methods

This retrospective cohort study included pregnant women with a history of cervical conization who visited before 20 weeks of gestation and gave birth at Osaka Women’s and Children’s Hospital, a tertiary perinatal center in Japan, between January 2013 and December 2022. The exclusion criteria were cerclage before 24 weeks of gestation, premature rupture of membranes (PROM) or delivery before 24 weeks of gestation, multiple pregnancies, fetal death, and major fetal anomalies. The clinical data of the study participants were extracted from their electronic medical records. This study was approved by the Institutional Review Board of Osaka Women’s and Children’s Hospital (approval number 1549-2, date: January 20, 2023). The requirement for informed consent was waived because of the retrospective nature of this study. 

At our hospital, prenatal management for pregnant women with a history of cervical conization is the same as that for women without such a history. Gestational age was primarily determined by early ultrasound [first-trimester crown-rump length (CRL)]. In cases where the last menstrual period (LMP) and CRL-based gestational age were in close agreement, LMP was used to confirm the estimated due date. The CL is measured at approximately 22 weeks of gestation in women without previous spontaneous PTB. Follow-up measurements were performed at the discretion of the physicians. Women with previous spontaneous PTB underwent fortnightly CL screening between 16 and 23 weeks of gestation. All CL measurements were performed by experienced obstetricians using transvaginal ultrasonography in a standard manner [9]. The shortest CL between 20 and 23 weeks of gestation was used in the analysis. Cervical cerclage was indicated for women with two or more histories of cervical insufficiency, a history of spontaneous PTB, shortened CL<25 mm before 24 weeks of gestation, and acute cervical insufficiency with visible fetal membranes before 26 weeks of gestation. We discussed with women with a previous spontaneous PTB about weekly intramuscular injections of 17-α hydroxyprogesterone caproate (17OHP-C) for prophylaxis. Prescriptions were provided according to the patient's preferences. Vaginal progesterone was not administered. 

The primary outcome was spontaneous PTB, followed by preterm labor or PROM before 37 weeks of gestation. The characteristics of the women, pregnancy course, and perinatal outcomes were compared between the two groups (spontaneous PTB vs. other births) using the Pearson chi-square or Fisher’s exact test for categorical variables and the Mann-Whitney U test for continuous variables. There were no missing data in this study; therefore, no imputation or case exclusion was necessary. Univariate logistic regression analyses were performed to examine the associations between the primary outcome and predictor variables, including obstetric history (previous second-trimester loss or spontaneous PTB), method of conception, body mass index before pregnancy, current smoking status, and CL. A multivariate prediction model was constructed using the backward selection procedure, initially considering variables with a univariate p-value<0.20. The study participants were stratified into groups according to independent predictors, and the incidence of spontaneous PTB in each group was calculated. Statistical significance was set at p<0.05. Statistical analyses were performed using SPSS 21.0 (IBM Corp., Armonk, NY, USA). Because the objective of this study was exploratory, the sample size was not predetermined.

## Results

We identified 164 pregnant women with a history of cervical conization during the study period. After exclusion (13 women underwent cerclage before 24 weeks of gestation, 7 women experienced PROM or delivered before 24 weeks of gestation, 17 women had multiple pregnancies, and 8 women had major fetal anomalies), 119 women were eligible for the analysis. Of these, 21(17.6%) had PTB before 37 weeks of gestation, 16 (13.4%) had spontaneous PTB, and 5 (4.2%) had medically indicated PTB. Spontaneous PTB before 34 weeks of gestation occurred in seven women (5.9%).

The baseline characteristics and pregnancy course of all study participants are shown in Table 1 and stratified according to pregnancy outcome.

**Table 1:** Characteristics of pregnant women with a history of cervical conization Values are presented as n (%) or median (interquartile range). BMI: body mass index; PTB: preterm birth; CL: cervical length; IVF: in vitro fertilization.

		Pregnancy outcome				
Characteristics	Total (n=119)	Spontaneous PTB (n=16)	Other births (n=103)	Statistical method	Test statistic	p-value
Maternal age, years	35 (32-38)	35 (33-38)	35 (32-38)	Mann-Whitney U	U=860.5	0.776
BMI before pregnancy, kg/m^2^	20.6 (19.1-22.5)	20.8 (18.1-22.2)	20.6 (19.2-22.5)	Mann-Whitney U	U=772.5	0.688
-BMI<18.5 kg/ m^2^	18 (15.1)	5 (31.3)	13 (12.6)	Fisher's exact	-	0.067
Multipara	59 (49.6)	8 (50.0)	51 (49.5)	Chi-square	χ²=0.001	1
Previous second-trimester loss or spontaneous PTB	9 (7.6)	4 (25.0)	5 (4.9)	Fisher's exact	-	0.019
-Second-trimester loss	1 (0.8)	0	1 (1.0)	-	-	-
-Spontaneous PTB	8 (6.7)	4 (25.0)	4 (3.9)	-	-	-
IVF	28 (23.5)	8 (50.0)	20 (19.4)	Fisher's exact	-	0.022
Current smoking status	22 (18.5)	4 (25.0)	18 (17.5)	Fisher's exact	-	0.493
Mid-trimester CL, mm	32.0 (25.9-38.0)	23.5 (19.4-27.0)	32.7 (28.3-38.3)	Mann-Whitney U	U=327.0	<0.001
-CL<25 mm	23 (19.3)	8 (50.0)	15 (14.6)	Fisher's exact	-	0.003

Of the nine women with a history of spontaneous PTB, four received prophylaxis with 17OHP-C. A woman underwent cerclage, as indicated by physical examination, at 24 weeks of gestation. Women who had a spontaneous PTB were more likely to have conceived by IVF and had a prior second-trimester loss, prior spontaneous PTB, and shorter mid-trimester CL than those who did not have a spontaneous PTB. Although not significant, there was a higher rate of being underweight before pregnancy in women with spontaneous PTB. Other variables were similar between outcome groups.

In the univariate analysis, obstetric history, conception by IVF, and shortened CL had p-values<0.20. A multiple logistic regression model of the spontaneous PTB was constructed using backward selection using these variables. Method of conception (adjusted OR for IVF: 4.5; 95% CI: 1.2-15.9; p=0.021) and CL (adjusted OR per 1 mm increment: 0.88; 95% CI: 0.82-0.94; p<0.001) remained significant variables (Table 2).

**Table 2:** Univariate and multivariate logistic regression analyses for spontaneous preterm birth *Adjusted for variables with a univariate p-value<0.20. OR: odds ratio; CI, confidence interval; PTB: preterm birth; BMI: body mass index; CL: cervical length; IVF: in vitro fertilization.

	Crude OR (95% CI)	p-value	Adjusted OR* (95% CI)	p-value
Previous second-trimester loss or spontaneous PTB	6.5 (1.5-27.7)	0.011	4.2 (0.84-20.7)	0.08
BMI before pregnancy per 1 increment	0.93 (0.78-1.11)	0.45	-	-
IVF	4.2 (1.4-12.4)	0.011	4.5 (1.2-15.9)	0.021
Current smoking status	1.6 (0.46-5.4)	0.47	-	-
Mid-trimester CL per 1 mm increment	0.89 (0.83-0.95)	<0.001	0.88 (0.82-0.94)	<0.001

**Figure 1:**
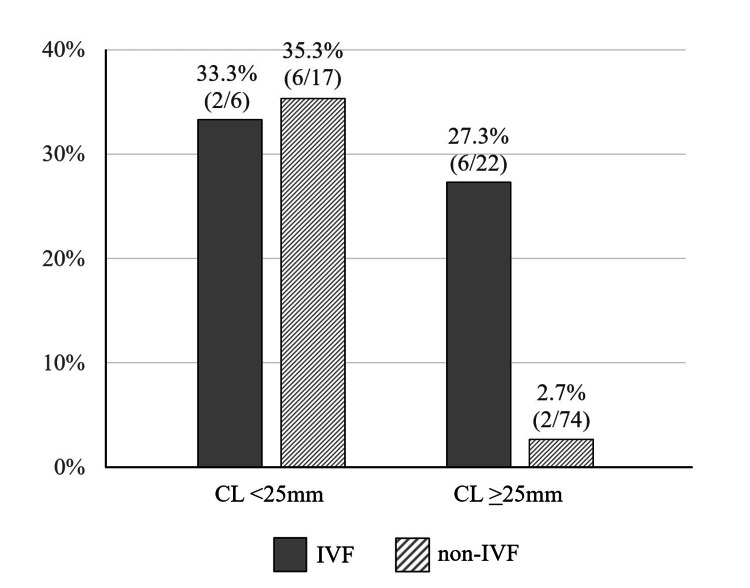
Rates of spontaneous preterm birth according to the independent predictors in 119 women with prior conization CL: cervical length; IVF: in vitro fertilization.

The study participants were divided into four groups according to two independent predictors of spontaneous PTB: method of conception and CL, with a threshold of 25 mm. The spontaneous PTB rates were 33.3% (2/6), 35.3% (6/17), 27.3% (6/22), and 2.7% (2/74) in IVF-conceived women with shortened CL, naturally conceived women with shortened CL, IVF-conceived women with normal CL, and naturally conceived women with normal CL, respectively (Figure 1).

When combining groups with either of the predictive factors, the incidence of spontaneous PTB was 31.1% (14/45). The sensitivity and specificity of the shortened CL alone were 50.0% and 85.4%, respectively. Assuming that either IVF or shortened CL was positive, the sensitivity and specificity were 87.5% and 69.9%, respectively.

## Discussion

In the present study, we found two independent predictors of spontaneous PTB among pregnant women with prior cervical conization: the method of conception and mid-trimester CL. The incidence of spontaneous PTB in women with a history of conization who conceived by IVF and/or had a shortened CL was approximately 30%, whereas those without IVF or shortened CL had a lower incidence of 3%.

The majority of previous studies on the predictors of PTB in pregnant women with prior cervical conization have focused on the surgical method, surgical specimen depth, and CL [5,8]. With regard to surgical variables, cold-knife conization seems to be the most hazardous method, and the risk of prematurity increases with increasing excisional specimen depth [8,10]. These results may guide surgeons to avoid cold-knife conization and preserve non-pathologic cervical tissue as much as possible; however, detailed surgical information, which may facilitate individual risk stratification, is not always obtainable for attending obstetricians in subsequent pregnancies. CL obtained by transvaginal ultrasonography in the mid-trimester is a known predictor of spontaneous PTB in pregnant women with prior cervical conization, as well as in the general population [3,5]. In 2004, Berghella et al. reported the incidence of spontaneous PTB before 35 weeks of gestation in pregnant women with prior cervical conization according to CL [5]. Among the 109 pregnant women who underwent prior cervical conization, 30 (27.5 %) had a short CL of <25 mm. The incidence of spontaneous PTB before 35 weeks of gestation in pregnant women with a shortened CL was higher than that in women with a longer CL (30.0% vs. 6.3%, relative risk 4.7, 95% CI: 1.6-15.3). Several additional studies have confirmed an association between a shortened CL and spontaneous PTB in pregnant women with prior conization [3,4,6,11]. Two studies reported the sensitivity and specificity of CL with a threshold of 25 mm for PTB before 37 weeks of gestation to be 41-75% and 95-98%, respectively [4,6]. The predictive value of CL in our study, with a sensitivity of 50.0% and specificity of 85.4%, was similar to that reported previously. 

A multivariate model, combined with other clinical variables, improved the predictive accuracy of CL for spontaneous PTB in the general obstetric population. Pils et al. assessed the association between CL values obtained by sequential transvaginal ultrasonography and other clinical variables and PTB before 34 weeks of gestation in women with prior cervical conization [12]. Using multivariate logistic analysis, they found that CL at 16 weeks of gestation and conception by IVF were independent predictors of PTB; however, the rate of exclusion due to an incomplete dataset exceeded 50%, which might warrant additional research. Our results confirm these findings.

Conception by IVF is a known determinant of PTB in the general obstetric population. According to a meta-analysis, singleton pregnancies after IVF are associated with a higher risk of PTB than spontaneous conception (risk ratio 1.54, 95% CI: 1.47-1.62) [13]. Using Finnish population-based data, Jakobsson et al. compared the incidence of PTB according to the method of conception in women who underwent cervical treatment, including conization (51.3%), ablation (40.2%), and other procedures (8.5%) [14]. The incidence of PTB was higher in women who underwent IVF than in those who did not undergo it (15.5% vs. 8.3%, p<0.01). Pinborg et al. assessed the risk of PTB according to plurality, method of conception, and history of dysplasia and conization using Danish population-based data [15]. In the cohort of singleton pregnancies and prior conization, the incidence of PTB in women who underwent IVF was higher than that in women who did not undergo it (13.1% vs. 7.5%, p-value not computed). The results of these two population-based studies, although not adjusted for CL, support our findings.

Our finding that conception by IVF and a shortened CL are independent predictors of spontaneous PTB in women with prior conization has clinical implications. The use of these two predictors may facilitate better risk stratification than the use of CL alone. Women who did not undergo IVF or without a shortened CL may not require further surveillance or intervention unless they are symptomatic. Women who underwent IVF or with a shortened CL may be considered to be at high risk for spontaneous PTB. Obstetricians should consider prescribing progesterone or offering cerclage placement to women with shortened CL. Although no preventative interventions targeting women who conceived by IVF have been established, patient education for those women on the avoidance of risk-increasing lifestyle habits and early symptoms of spontaneous PTB may be worth recommending. Future investigations are warranted to establish effective interventions to prevent spontaneous PTB in these women.

Our study had several limitations. First, the sample size was limited because this was a retrospective, single-center study. Large-scale multicenter studies would allow researchers to draw more robust conclusions regarding the predictors of spontaneous PTB in pregnant women with prior conization. Second, detailed information on the surgical procedure was not available; therefore, we could not include the methods of conization and excisional depth in the analysis. Third, the generalizability of our findings may be limited, as the study population consisted exclusively of Japanese women with a relatively low mean BMI compared to international populations [16]. Fourth, although cerclage was indicated according to predefined criteria, clinical judgment was inevitably involved in decision-making, which may have introduced indication bias. Fifth, progesterone use was not standardized; only four of nine eligible women received 17OHP-C, and vaginal progesterone was not administered. Sixth, the interval between conization and subsequent pregnancy and data on cervicovaginal infections, both known risk factors for preterm birth [17,18], were not systematically available, preventing us from including these variables in the analysis. Seventh, our study did not include long-term neonatal outcomes. Finally, while ultrasound measurements were performed by experienced obstetricians according to standard protocols, inter-operator variability cannot be fully excluded. However, the consistent clinical management throughout the study period and the specific gestational age at CL measurement were the strengths of our study, which may allow us to generalize our results to similar institutions.

## Conclusions

We found that conception by IVF and a shortened mid-trimester CL are significant predictors of spontaneous PTB among pregnant women with prior cervical conization. Risk stratification using these predictors may improve the sensitivity of the spontaneous PTB prediction. Further research is needed to validate our findings and develop preventative therapies for at-risk women with a non-shortened CL.
